# Influence of Methods of Corn Starch Modification and Used Sweetener on the Functional Properties of Blackberry Jelly-like Dessert

**DOI:** 10.3390/molecules28020498

**Published:** 2023-01-04

**Authors:** Marzena Włodarczyk-Stasiak, Artur Mazurek, Radosław Kowalski

**Affiliations:** Department of Analysis and Evaluation of Food Quality, Faculty of Food Science and Biotechnology, University of Life Sciences in Lublin, Skromna Street 8, 20-704 Lublin, Poland

**Keywords:** blackberry jelly-like dessert, clean label, diet product, modified starch, physical properties

## Abstract

A study was conducted on selected physicochemical properties of blackberry jelly-like desserts (kissel) prepared from physically modified starches (with various degrees of inhibition) and chemically modified starches (with various degrees of cross-linking). The desserts were conventionally sweetened with saccharose (S) or, as a dietary alternative, xylitol (X). The characteristics of changes in the viscosity of the kissels as a function of temperature and time were determined. It was noted that regardless of the sweetener used, the viscosity of the kissels increased with the decreasing degree of inhibition (high < medium < low). Regardless of the kind of modification of the starch used for the preparation of the kissels and of the kind of sweetener, thixotropy was observed. Desserts prepared from inhibited starch with xylitol (CL + X) were characterised by the biggest range of their hysteresis loop. Progressing retrogradation was noted with the decrease in the temperature of the experiment (+20 °C and +4 °C). After 7 days of storage, kissels sweetened with saccharose were characterised by a low transparency, which may indicate retarded retrogradation; however, on day 28, the transparency significantly increased, exceeding the values of transmittance for samples sweetened with xylitol. The tendency towards syneresis was tested at +4 °C and −22 °C. The substitution of saccharose with xylitol only caused a slight modification of viscosity. Regardless of the sweetener used and of the level of starch inhibition, lower ranges of the hysteresis loop were noted (apart from CL + X) than in the case of kissels obtained from chemically modified starches. Distinctly lower values of kissel “aging” indices were noted in the case of samples obtained from inhibited starches, and their colour did not significantly differ in relation to the dessert prepared from native starch.

## 1. Introduction

Starch is a fundamental component of carbohydrate food products. It is used for the thickening of sauces, soups, sweet puddings, jelly-like desserts (kissel) and many other processed foods. The use of native starches in the food industry is rather limited, which has led to ongoing research aimed at the obtainment of starches with new, universal functional properties that will satisfy food producers, and products with their admixture will meet the expectations of consumers. Modified starches display considerably higher temperature stability during heating, cooling, freezing and thawing relative to their native counterparts. This allows for the creation of products with constant quality determinants and high taste values during product turnover, i.e., in storage (under refrigeration or freezing) and transport [1].

Producers of food additives more and more frequently offer physically modified starches, conforming with the “clean label” idea, as substitutes for the chemically modified starches that enjoyed extreme popularity for decades. Starches obtained through physical modification more and more often attain the same level of application universality as that of their chemically modified counterparts [2,3].

The innovative aspect of this study is the analysis of the interactions and use of “inhibited starch” in food systems. The conditions of the modification involve the effect of high temperature on starch granules in a dehydrated environment. However, these are not the classic conditions of hydrothermal modification (in the solid phase or in the form of paste), as the modification in this case is conducted in alcohol solutions (>80%) under atmospheric pressure or elevated pressure. The conditions of the modification and the possibility of their modelling (time, temperature, and alcohol concentration) do not cause any degradation of starch granules or their gelatinization, so the starch retains its granular and “hardened” structure, which is more resistant to culinary thermal treatment than native starch. Such starches are obtained through physical modification under the effect of high temperature at a reduced water availability. This allows for the retention of the granular structure, and the products of such a modification are inhibited starches [4]. These starches can perform the function of a non-chemically modified starch thickener with properties similar to those of starch thickeners obtained through chemical modification. Inhibited starches can be used in food products subjected to mild and drastic heat treatment during pasteurisation or sterilisation (UHT), in which product stability is required over a broad range of temperatures in the course of refrigeration, freezing and heating [5].

The available literature of the subject only provides reports concerning the properties of inhibited starches [4]. There have been reports on research concerning interactions with food components (proteins, fats, and sugars), the effect of inhibited starches on the properties and shelf life of food products, and comparisons of inhibited starches with chemically modified starches.

When designing a new product, it worth considering the substitution of popular sweeteners (saccharose and glucose-fructose syrups) with natural ones such as beech sugar (xylitol). Temperature sensitivity, specific taste, colour, and allergising properties often disqualify honey as a sweetener. The physicochemical, sensory and health-promoting properties of xylitol mean that it can be a good substitute for saccharose, as its sweetness is identical, its calorific value is 40% lower, and its glycaemic index is 10-fold lower [6,7,8,9].

This study was focused on the analysis of selected physicochemical properties of blackberry jelly-like desserts prepared from chemically or physically modified corn starches in which saccharose was substituted with xylitol without any significant quality changes. The realization of this research objective involved the analysis of the rheological properties of the desserts (flow curves and viscosity curves in the gradient of temperature and time), changes taking place during long-term storage in drastic temperature conditions (gel syneresis and clarity), and changes in the index that determines the acceptability of blackberry jelly-like desserts for consumers (colour in the L*a*b* system).

## 2. Results and Discussion

### 2.1. Rheological Properties

**Figure 1:**
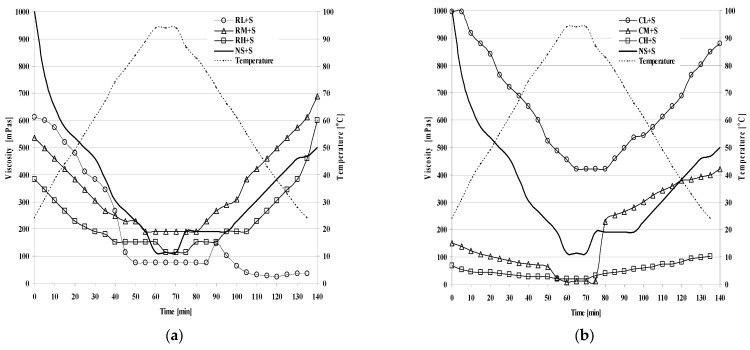
Viscosity curves of blackberry jelly-like desserts obtained from chemically modified corn starch sweetened with sucrose (**a**) or xylitol (**b**).

**Figure 2:**
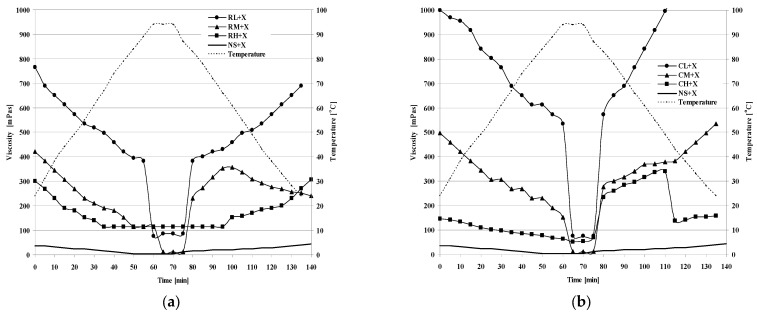
Viscosity curves of blackberry jelly-like desserts obtained from physically modified corn starch sweetened with sucrose (**a**) or xylitol (**b**).

The viscosity of starch gels with the addition of sweetened blackberry fruits was measured as a function of temperature and time (viscosity vs. temperature and viscosity vs. time). The shapes of the viscosity change curves fundamentally differed for the kissels obtained from physically and chemically modified corn starches (Figure 1 and Figure 2). The shapes of the curves were affected by the method and degree of starch modification, as well as by the properties of the sweetener used. The process of heating (1 °C/min) produced a decrease in viscosity in all analysed gels and, in most of the samples, maintaining a temperature of ca. 96 °C contributed to the attainment of the lowest values of viscosity (Table 1). It was also observed that with increases in the degree of cross-linking (RL + S < RM + S < RH + S; RL + X < RM + X < RH + X) and the degree of inhibition (CL + S < CM + S < CH + S; CL + X < CM + X < CH + X), the viscosity of the blackberry jelly-like desserts decreased. A similar relationship between gel viscosity decrease and increase in the degree of the cross-linking of chemically modified starches was demonstrated by Włodarczyk-Stasiak et al. [10]. In a subsequent study, Włodarczyk-Stasiak et al. [4] addressed the definition of the properties of inhibited starch as viscoelastic, similar to the properties of chemically cross-linked starches. Based on the results of their study, the authors claimed that inhibited starches have similar rheological properties to those of chemically modified starches, which allows for similar applications and prediction of the “shelf life” of products with their admixture [5].

Regardless of the sweetener used, gels obtained from inhibited starches were characterised by a lower initial viscosity compared with their final viscosity. This was attributed to the aggregation of starch chains with the possibility of the recreation of their three-dimensional network [11].

It was also demonstrated that in the case of samples with low degrees of cross-linking (RL + S, RL + X) and low inhibition (CL + S, CL + X), the highest level of viscosity was attained over the entire range of measurement, regardless of the sweetener used. A probable cause of this is the reduction in the water-plastifying effect and the increased ease of the formation of sugar bridges between starch chains in amorphous areas of starch, which inhibits the penetration of water molecules and stabilises newly formed structures [12,13,14,15,16]. Probable starch–sugar interactions were mentioned by Chang [17], who attributed them to the effect of the sugar bridge, the antiplastifying role of sugar, and competition for water molecules as co-solvents. The cross-links forming between starch chains and sugar in amorphous regions of starch can play a stabilising role, enhancing viscosity or retarding gelatinisation [18,19].

#### Thixotropy

The phenomenon of thixotropy is related to shear thinning (mixing) and can be attributed to the aggregation of particles in the unstressed state, which causes the formation of an internal suspension structure and the partial or total disintegration of the formed structure of a given non-Newtonian fluid subsequently subjected to shear [20]. The opposite phenomenon to thixotropy is anti-thixotropy, which consists of the shear thickening of fluids. This phenomenon is attributed to structural changes in fluids, the consequence of which is increased internal resistance; therefore, shear forces lead to the development of the internal structure of fluids [21].

**Figure 3:**
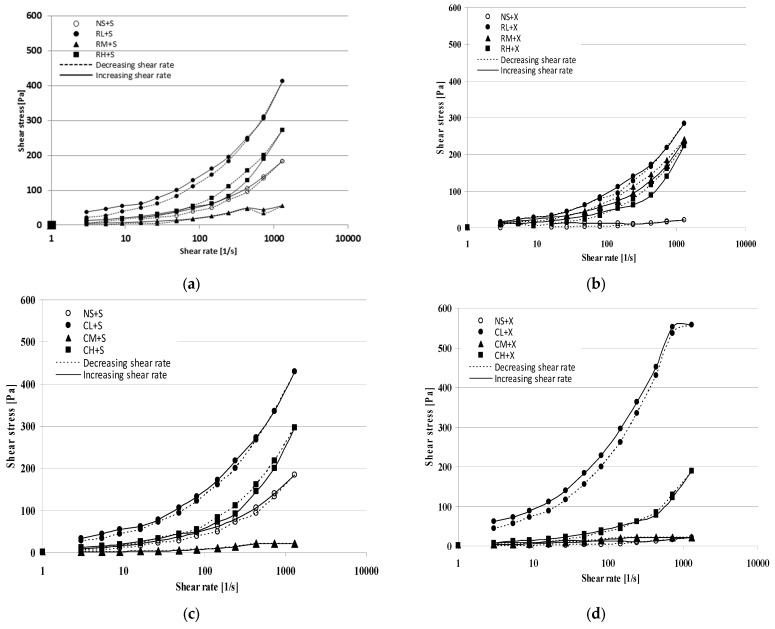
Thixotropy of blackberry jelly-like desserts obtained from modified corn starches sweetened with sucrose (**a,c**) or xylitol (**b,d**).

Figure 3 presents the flow curves of blackberry kissels produced on the basis of chemically modified (Figure 3a,b) and inhibited starches (Figure 3c,d) sweetened with sucrose or with xylitol. For all analysed samples, different flow curve shapes were observed, determined at increasing and decreasing shear rates relative to the shear stress values. The area formed between the curves is defined as hysteresis. Its value provides valuable information on the direction of changes in the kissels over the course of their preparation for consumption and storage. If the flow curve determined at increasing shear rates is situated beneath the curve plotted at decreasing shear rates, the value of hysteresis is positive. Such a rheological characteristic indicates that a sample is thinned under the effect of shear, while negative values of hysteresis indicate the thickening of samples (Table 2). It is also worth noting the effect of the sweetener used on the range of hysteresis. In the case of kissels obtained from inhibited starches sweetened with xylitol (CL + X, CM + X, CH + X), the size of the hysteresis loop was considerably bigger than in the case when sucrose was used as the sweetener. This can be attributed to the stronger gelatinising properties of the starch–xylitol system, the structure of which is destroyed under increasing shear stress, and the time of relaxation was shown to be insufficient for its recreation. The blackberry kissels obtained from chemically modified starches were characterised by larger areas of hysteresis when saccharose was used as the sweetener.

Sikora et al. [22] described hysteresis as an indicator of the degree of structural damage due to shear—the smaller the area of the hysteresis loop, the lower the degree of structural damage under shear. Those authors also indicated a key impact of the content of saccharose in the system on structural stability [22]. It was demonstrated that at 0–20% of saccharose, there were no greater differences between loop sizes, while the size of the hysteresis loop significantly decreased at a 30% saccharose concentration.

Basically, thixotropy is a measure of the instability of systems, and it is defined as an unfavourable effect, hence the continued research on physical, chemical or biochemical modifications of starch that will contribute to a reduction in the area of the hysteresis loop [23].

### 2.2. Retrogradation and Syneresis as a Measure of Ageing of Blackberry Jelly-like Desserts

Retrogradation is an undesirable phenomenon that causes a change in the texture and stability of food products. The factors that determine this phenomenon include the botanical variety of starch, temperature and storage time. The determination of the degree of clouding through the measurement of transmittance provides information on the intensity of starch retrogradation. In the course of retrogradation, the spaces between starch chains (amylose and amylopectin) decrease. Starch transforms from a partially amorphous state to an ordered form, with semi-crystalline networks, that is observed as separation of the product into layers. In the case of liquid products, one can observe a “sediment” forming on the bottom of the vessel, above which the clarity of the liquid increases [24,25,26].

**Figure 4:**
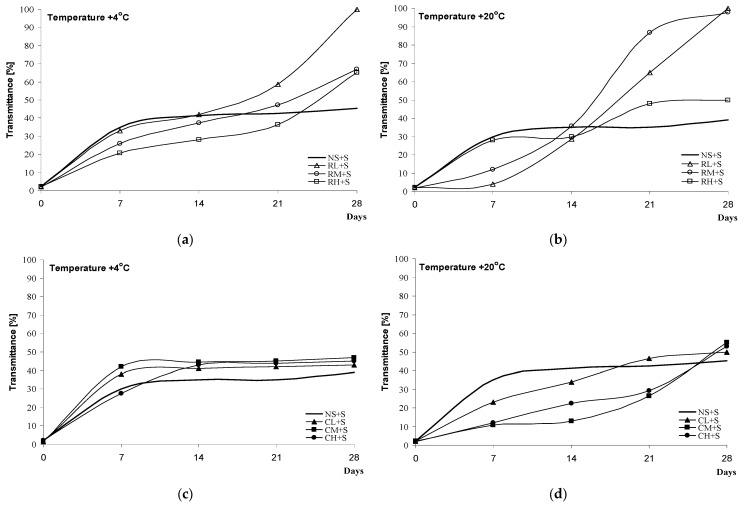
Retrogradation of black jelly-like desserts prepared on the basis of chemically (**a**,**b**) or physically (**c**,**d**) modified starches sweetened with sucrose.

**Figure 5:**
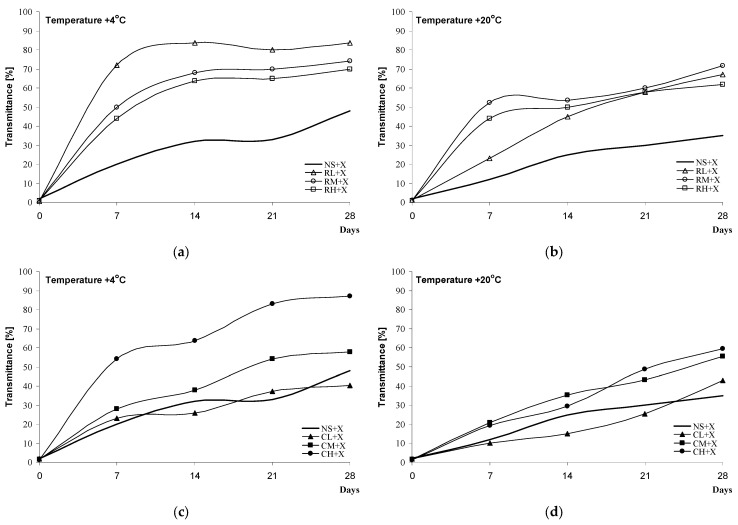
Retrogradation of black jelly-like desserts prepared on the basis of chemically (**a**,**b**) or physically (**c**,**d**) modified starches sweetened with xylitol.

Here, the intensity of retrogradation was determined by measuring the transmittance of the blackberry jelly-like desserts at +4 °C (Figure 4a,c and Figure 5a,c) and +20 °C (Figure 4b,d and Figure 5b,d). Higher values of transmittance (%) indicate an intensification of retrogradation. Regardless of the method of starch modification used for the preparation of the blackberry jelly-like desserts, the samples were characterised by higher values of transmittance (%) at +4 °C than the same samples at +20 °C. This observation is supported by a study by Włodarczyk-Stasiak et al. [27] on the retrogradation of gels of waxy corn starch, which demonstrated a more intensive rate of the process at lower temperatures.

The shapes of the curves illustrating the progress of retrogradation differed in relation to the kind of sweetener used. Blackberry jelly-like desserts sweetened with saccharose were characterised by fairly low values of transmittance (%) after 7 days compared with samples sweetened with xylitol. In spite of the initially (after the 7th day) intensive retrogradation of the samples with xylitol, a significant slow-down of the process was noted over subsequent days (14, 21, and 28). Regardless of the kind of sweetener used, the blackberry jelly-like desserts obtained from inhibited starch had lower values of transmittance (%) compared with those sweetened with saccharose.

The process of starch retrogradation under the effect of added sugars has been extensively researched, but the obtained results are not coherent. Marsh et al. [24] argued that saccharose and maltose delayed the retrogradation of wheat gels, fructose intensified it, and xylose downright inhibited the process of the recrystallisation of amylopectin, whereas Cairns [28] demonstrated that glucose slowed down the process while saccharose and ribose inhibited the retrogradation of gels obtained from wheat starch. Prokopowicz et al. [29], on the other hand, indicated an intensification of retrogradation with the participation of glucose and fructose and a slow-down of the process when gels of waxy corn starch contained ribose or xylose. Farhat et al. [30] studied the effect of sugars on the rate of the retrogradation of gels obtained from waxy maize starch. Researchers proved that, apart from the kind of sugar, the availability of water plays a key role in the process. A 30% content of fructose, saccharose, or xylose in samples at a water content of 24 ± 1% caused an increase in the rate of retrogradation in the sequence of xylose < saccharose < fructose, while at a water content of 35 ± 1%, the addition of xylose resulted in a significant slow-down of retrogradation. On the other hand, Dongryel Yoo et al. [31] arrived at a different conclusion regarding the content of sweetener and water availability. In the cited study, the authors analysed the rate of the retrogradation of rice gels with 10%, 20% and 30% saccharose, and they observed that the higher the concentration of saccharose, the slower the rate of retrogradation. In support of their study, they referred to the results presented in [29,32,33].

**Figure 6:**
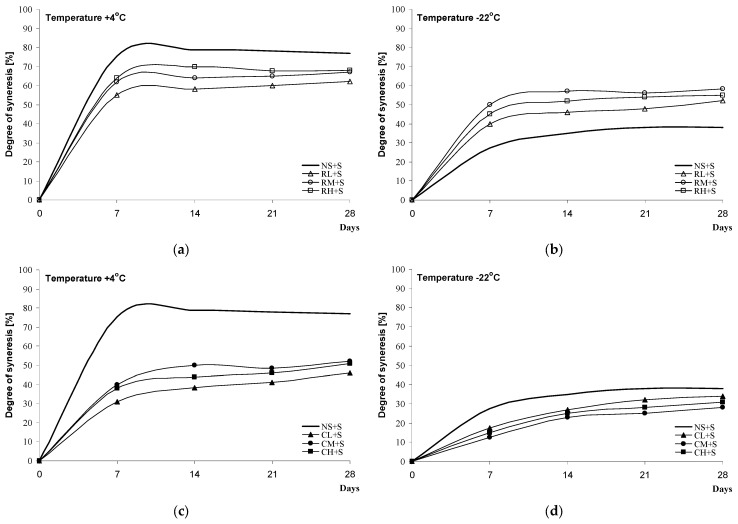
Syneresis of black jelly-like desserts prepared on the basis of chemically (**a**,**b**) or physically (**c**,**d**) modified starches sweetened with sucrose.

**Figure 7:**
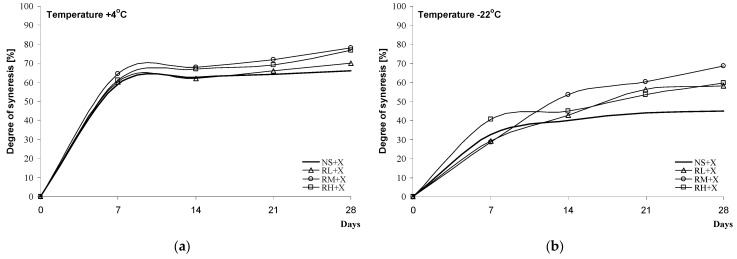

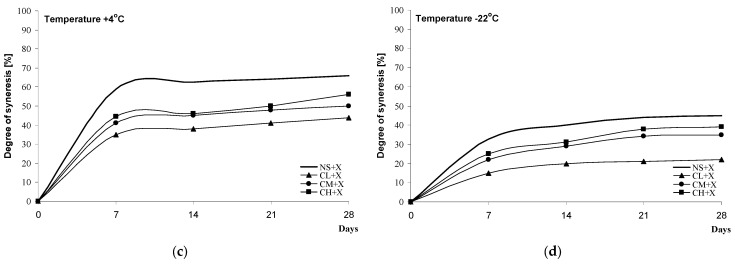
Syneresis of black jelly-like desserts prepared on the basis of chemically (**a**,**b**) or physically (**c**,**d**) modified starches sweetened with xylitol.

Measurements of the degree of syneresis of blackberry kissels can be an indicator of starch (amylopectin) tendency towards the liberation of water as a consequence of retrogradation. Here, the susceptibility of blackberry kissels to syneresis was analysed under refrigeration conditions at +4 °C (Figure 6a,c and Figure 7a,c) and after deep freeze at −22 °C (Figure 6b,d and Figure 7b,d). Distinct differences in the syneresis values were noted in relation to the method of the modification of the starch used for the preparation of the kissels. Desserts obtained on the basis of chemically modified starch sweetened with saccharose, with various degrees of cross-linking, were characterised by syneresis at levels of 60–79% at +4 °C and 49–55% at −22 °C, i.e., values close to or higher than those obtained for native starch (Figure 6a,b). Much higher stability and therefore smaller seepage were characteristic of kissels based on inhibited starch, the values for which were 43–50% at +4 °C and 24–30% at −22 °C (Figure 6c,d). No correlation was noted between the degree of substitution/level of inhibition and the value of syneresis. When xylitol was used as the sweetener, slight decreases in syneresis were noted as follows: 9% at +4 °C and 6% at −22 °C for samples with chemically modified starch (Figure 7a,b) and 6% at +4 °C and 4% at −22 °C in for kissels with inhibited starch (Figure 7c,d). In the case of samples from inhibited starch and xylitol, it was noted that regardless of the temperature at which the kissels were kept (+4 °C or −22 °C), syneresis decreased with decreases in the level of inhibition (CL + X < CM + X <CH + X).

In the context of the current research, available reports have primarily considered the analysis of the botanical origins of starch and its susceptibility to syneresis. Gel-like, spongy, layered structures of de-frozen maize and potato sauces with a flaking tendency were mentioned by authors such as [1,34], while sauces from rice or waxy starch, after defreezing, have been characterised by the structure of a freshly prepared sauce.

### 2.3. The Effect of Sweetener on the Colour of Blackberry Kissels

The perception of the colour of food products often has a bearing on their acceptance or rejection. Among the analysed samples, the kind of sweeter used had a strong differentiating effect. An approximately 11% increase in L* and an approximately 50% increase in a* were noted for samples with the addition of xylitol (Table 3).

The analysis of ΔE revealed that the gels obtained from inhibited starches only slightly differed from the reference samples (native starch), as the range of ΔE values was from 0.88 to 1.88, due to which the colour differences could be described as undetectable or slight. Notably greater differences were noted for samples obtained from chemically modified starches, for which the values ranged as 1.26 < ΔE < 4.45—which indicates colour differences that are distinct and observable by consumers relative to the control.

A distinct change in the colour of maize gels (−a*) compared with wheat gels (+a*) was mentioned by Martínez et al. [35]. Those authors pointed to the presence of saccharose and polyols (sorbitol and xylitol) as factors that significantly reduced the luminance of the gels (L*) while simultaneously increasing the value of a*.

Desserts prepared on the basis of physically modified starches with varying degrees of inhibition show similar or better stability compared to desserts obtained from chemically modified starches. Such satisfactory results suggest that physically modified starches can also be used to stabilise, thicken, and add texture to other food products, such as sauces, ketchups and creams. However, due to the differences in the qualitative composition of these products, the use of inhibited starches requires further research.

## 3. Materials and Methods

### 3.1. Materials

The thickening material used in this study consisted of physically and chemically modified commercial corn starches acquired from the company TATE & LYLE. The method of preparation and the properties of the starches were described by Włodarczyk-Stasiak [4,27,36]. The substances used as sweeteners in the preparation of the desserts were saccharose (from “Polski Cukier”) and xylitol (from Ksylitol Danisco Finland). The fruit component was blackberry from the Lublin region of the 2022 autumn harvest (Table 4).

### 3.2. Preparation of the Desserts

The quantitative and qualitative composition of the desserts is presented in Table 5.

Blackberry fruits with the sweetener and water (40 mL) were brought to the boiling point and then kept at a temperature of 100 °C for 5 min with constant stirring. The obtained extract was separated and mixed with a prepared starch gel obtained by heating (90–100 °C for 6 min) modified starches (3.2 g) with the remaining volume of water (20 mL). The dessert prepared in this manner was cooled down to 24 °C and then subjected to analysis. 

### 3.3. Methods

#### 3.3.1. Rheological Properties

The flow curves and the measurements of viscosity in the gradient of temperature and time were determined using a Rheotest 2 tester (VEB MLW Prüfgeräte-Werk Medingen, Germany).

##### Change of Viscosity of Blackberry Kissels in the Gradient of Temperature and Time

Measurements of the viscosity of the modified starch gels were performed according to the Winkler method after certain necessary modifications [37]. The viscosity characteristics of the blackberry jelly-like desserts were determined in a S/S-1 cylinder (25 mL) by heating/cooling down within a temperature range from 20 °C to 96 °C (1 °C/min), keeping them for 20 min at Tconstant = 96 °C. Viscosity measurements were taken at 5 min intervals at a shearing rate of 145.8 (1/s), which corresponded to a rotational speed of 25 rpm.

##### Flow Curves of Blackberry Jelly-like Desserts at Increasing and Decreasing Shear Rates

The flow curves of the blackberry kissels were determined using the Rheotest 2 tester. The determinations were conducted using the S/S-1 set of measuring cylinders with sequence “a” (12 measurement points). The results were taken at 1 min intervals at increasing and decreasing shear rates. The shearing stress τ (Pa) was calculated from the product of the value of α and the constant “Z” (Pa) for the given set of cylinders.

The obtained results were used to plot the graphs of shear stress versus shear rate.

#### 3.3.2. Functional Properties

##### Paste Clarity

The blackberry jelly-like desserts were stored at +20 °C or +4 °C for 4 weeks. Measurements of transmittance (T) were taken at 7-day intervals at λ = 650 nm and a temperature of 20 °C, referenced to distilled water. Samples stored at +4 °C were pre-measured and then heated for about 90 min to +20 °C [37].

##### Degree of Syneresis

The blackberry jelly-like desserts were stored for 4 weeks at −22 °C or +4 °C. The degree of syneresis was determined from the volume of liberated water (mL/10 mL gel; %) after centrifugation (3000 rpm, 10 min). Measurements were taken at 7-day intervals at 20 °C [37].

### 3.4. Colour Measurement in the L*a*b System

The analysis of the colour of the obtained blackberry kissels was conducted with an NH310 colour tester and the use of the CIE LAB (L*a*b*) method.

The measurements were taken at room temperature (20 °C), changing the position of the cuvette with the samples ten times. Averaged colour parameters were used to calculate the total colour difference according to Formula (1).

The results were processed based on the criterion according to which the absolute colour differences ΔE form the following ranges:

(1) $$\Delta E=\sqrt{\left(\Delta {\;L}^{2}\right)+\left(\Delta {\;a}^{2}\right)+\left(\Delta {\;b}^{2}\right)\;}$$

ΔE 0–1—undetectable differences.

ΔE 1–2—small differences.

ΔE 2–3.5—medium, detectable differences.

ΔE 3.5–5—distinct differences.

ΔE above 5 means big differences in the hue of the colour.

### 3.5. Statistical Analysis

The reported data are expressed as the average of triplicate observations. In the study, the mean values (*x*) and the standard deviation (σ) were calculated from the range (*x*–2σ; *x* + 2σ); data out of this range were rejected. An ANOVA was used to calculate significant differences in treatment means and LSD (*p* < 0.05).

## 4. Conclusions

The authors of this study analysed selected physicochemical properties of blackberry jelly-like desserts (kissels) obtained from inhibited and chemically modified (cross-linked) starches that were sweetened with saccharose or xylitol. The results of the analyses did not provide a clear-cut indication of which variables (method of starch modification or kind of sweetener used) produced a stronger differentiation of the physicochemical properties of the analysed blackberry kissels. The use of inhibited starches (which, due to the method and extent of modification, can be denoted as starch on the product label) resulted in the obtainment of products with similar viscosity change characteristics to their chemically modified counterparts. The substitution of saccharose with xylitol only caused a slight modification of viscosity. Regardless of the sweetener used and the level of starch inhibition, lower ranges of the hysteresis loop were noted (apart from CL + X) than in the case of kissels obtained from chemically modified starches. Distinctly lower values of kissel “ageing” indices were noted in the case of samples obtained from inhibited starches, and their colour did not significantly differ in relation to the dessert prepared from native starch.

In view of the above results, one can state with complete conviction that the use of physically modified (inhibited) starch is fully acceptable in the preparation of desserts with diverse composition (in terms of the sweetener used) destined for long-term storage within a broad range of storage temperatures. The use of xylitol as a substitute for saccharose (without any major changes in product quality) allows for such a product to be defined as dietary, thus conforming to the clean label idea.

## Figures and Tables

**Table 1:** Parameters of the viscosity change of blackberry jelly-like desserts.

Code	Minimum Viscosity, η_max_ (mPa∙s)	Temperature at Minimum Viscosity, t η_max_ (°C)	Viscosity at 96 °C, η_96 °C_ (mPa∙s)	Viscosity after 20 min of Storage at 96 °C, η_96 °C/20′_ (mPa∙s)	Viscosity to 20 °C, η_20 °C_ (mPa∙s)	
**with Sucrose**					**Start**	**Finish**
**NS + S**	114.81 ^b^	94 ^a^	114.81 ^b^	114.81 ^b^	1000 ^d^	500
**CL + S**	420.98 ^c^	94 ^a^	420.98 ^c^	420.98 ^d^	995 ^d^	880
**CM + S**	3.95 ^a^	90–95 ^a^	3.95 ^a^	225.5 ^c^	167 ^b^	420
**CH + S**	19.78 ^a^	85–94 ^a^	19.78 ^a^	30.65 ^a^	65 ^a^	103
**RL + S**	23.74 ^a^	95 ^a^	76.54 ^a^	76.54 ^a^	612 ^c^	35
**RM + S**	191.35 ^b^	85–94 ^a^	191.35 ^b^	191.35 ^bc^	535 ^c^	688
**RH + S**	114.81 ^b^	94 ^a^	114.81 ^b^	114.81 ^b^	382 ^bc^	600
**with Xylitol**					**start**	**finish**
**NS + X**	3.95 ^a^	85–94 ^b^	3.95 ^a^	3.95 ^a^	35 ^a^	43 ^a^
**CL + X**	76.54 ^b^	94 ^b^	76.54 ^b^	76.54 ^b^	1000 ^e^	1530 ^e^
**CM + X**	11.87 ^a^	94 ^b^	11.87 ^a^	11.87 ^a^	497 ^d^	535 ^c^
**CH + X**	51.44 ^b^	94 ^b^	51.44 ^b^	51.44 ^b^	146 ^b^	158 ^b^
**RL + X**	76.54 ^b^	90 ^b^	87.06 ^bc^	87.06 ^bc^	765	720 ^d^
**RM + X**	11.87 ^a^	94 ^b^	11.87 ^a^	11.87 ^a^	420 ^cd^	240 ^b^
**RH + X**	114.81 ^c^	60–70 ^a^	114.81 ^c^	114.81 ^c^	300 ^c^	306 ^bc^

The same letters in columns indicate values that are not significantly different at α = 0.05.

**Table 2:** Hysteresis loop size of blackberry jelly-like desserts.

Hysteresis Loop Area							
**with Sucrose**	**NS + S**	**CL + S**	**CM + S**	**CH + S**	**RL + S**	**RM + S**	**RH + S**
	−66	−92	+4	+52	−140	−42	+61
**with Xylitol**	**NS + X**	**CL + X**	**CM + X**	**CH + X**	**RL + X**	**RM + X**	**RH + X**
	−57	−251	−2	+34	−39	+46	−25

**Table 3:** Measurements of the colour of blackberry kissels (L*, a*, b*).

Code	L*	a*	b*	ΔE
**with Sucrose**				
**NS + S**	26.6 ± 0.03 ^a^	2.1 ± 0.04 ^b^	0.10 ± 0.02 ^a^	-
**CL + S**	26.13 ± 0.01 ^a^	1.34 ± 0.01 ^a^	0.05 ± 0.02 ^a^	0.89
**CM + S**	26.02 ± 0.11 ^a^	1.49 ±0.01 ^a^	0.06 ± 0.01 ^a^	0.84
**CH + S**	25.69 ± 0.16 ^a^	1.55 ± 0.02 ^a^	0.07 ± 0.01 ^a^	1.06
**RL + S**	25.04 ± 0.41 ^a^	1.00 ± 0.02 ^a^	0.00 ± 0.01 ^a^	1.91
**RM + S**	25.93 ± 0.05 ^a^	1.04 ± 0.01 ^a^	−0.03 ± 0.01 ^ab^	1.26
**RH + S**	24.30 ± 0.62 ^a^	0.87 ± 0.02 ^a^	−0.09 ± 0.02 ^ab^	2.79
**with Xylitol**				
**NS + X**	29.79 ± 0.05 ^c^	2.77 ± 0.02 ^b^	0.09 ± 0.01 ^a^	-
**CL + X**	29.13 ± 0.03 ^ab^	1.01 ± 0.01 ^a^	0.07 ± 0.01 ^a^	1.88 ^a^
**CM + X**	28.78 ± 0.41 ^ab^	1.24 ± 0.01 ^a^	0.10 ± 0.02 ^a^	1.83 ^a^
**CH + X**	28.51 ± 0.33 ^ab^	1.99 ± 0.02 ^ab^	0.22 ± 0.01 ^b^	1.50 ^a^
**RL + X**	26.91 ± 0.45 ^a^	1.12 ± 0.01 ^a^	0.00 ± 0.01 ^a^	3.32 ^b^
**RM + X**	26.86 ± 0.19 ^a^	1.46 ± 0.01 ^a^	0.06 ± 0.02 ^a^	3.21 ^b^
**RH + X**	25.45 ± 0.56 ^a^	1.80 ± 0.02 ^ab^	0.10 ± 0.01 ^a^	4.45 ^c^

The same letters in columns indicate values that are not significantly different at α = 0.05.

**Table 4:** Characteristics of modified corn starches and chemical composition of desserts.

Commercial Name	Type of Modification	Degree of Cross-Linking	Code	
			with Sucrose	with Xylitol
Waxy corn starch	-	Native	**NS + S**	**NS + X**
Claria Essential	Physical (inhibition)	Low	**CL + S**	**CL + X**
Claria Plus		Medium	**CM + S**	**CM + X**
Claria Elite		High	**CH + S**	**CM + X**
Resistamyl 341	Chemical (substitution and crosslinking)	Low	**RL + S**	**RL + X**
Resistamyl 347		Medium	**RM + S**	**RM + X**
Resistamyl 342		High	**RH + S**	**RH + X**

**Table 5:** Quantitative and qualitative composition of the desserts (g/100 g).

Water	Starch Material (Physical or Chemical Modified Corn starch)	Sweetener (Sucrose or Xylitol)	Fruit (Blackberry)
g/100 g			
60.6	3.2	3.9	32.3

## Data Availability

Not applicable.
